# Editorial: Advances in Molecular Docking and Structure-Based Modelling

**DOI:** 10.3389/fmolb.2022.839826

**Published:** 2022-01-27

**Authors:** Joseph Rebehmed, Alexandre G. de Brevern, Ramanathan Sowdhamini, Agnel Praveen Joseph

**Affiliations:** ^1^ Department of Computer Science and Mathematics, Lebanese American University, Beirut, Lebanon; ^2^ Université de Paris and Université de la Réunion, INSERM UMR_S 1134, DSIMB, Paris, France; ^3^ National Centre for Biological Sciences (TIFR), Bangalore, India; ^4^ Molecular Biophysics Unit, Indian Institute of Science, Bangalore, India; ^5^ Institute of Bioinformatics and Applied Biotechnology, Bengaluru, India; ^6^ Scientific Computing, Science and Technology Facilities Council, Research Complex, Rutherford Appleton Laboratory, Didcot, United Kingdom

**Keywords:** docking, molecular dynamics simulation, structure-function relationship, structure modeling, drug design

**Editorial on the Research Topic**

**Advances in Molecular Docking and Structure-Based Modelling**

The three-dimensional (3D) structures of proteins form the structural framework of their functions. Having access to the structure allows scientists to better apprehend molecular details of protein functions; it is also crucial for protein engineering, e.g., to modify and optimize an enzyme for a certain biochemical reaction; or for designing new and improved drug molecules based on the structure of the target protein. Structures are also needed to investigate how proteins interact; a vast majority of the protein-protein interface residues are involved in extensive intra-protein interactions apart from inter-protein interactions (Jayashree et al., 2019).

With the increase in protein structures available in the Protein DataBank (PDB, 184,929 entries, on the 11th of December 2021, https://www.rcsb.org) (Berman et al., 2000), and the recent development of machine learning approaches for protein structure prediction, *e.g.* AlphaFold2 (Jumper et al., 2021), it is evident that protein structures will be an essential component of a large number of biological research studies. It also highlights the importance of efficient computational tools to process the structures and derive various biological interpretations.

These *in silico* methodologies are often complex, have limitations, and the results must be associated with appropriate statistical and quality measures. The objective of this Research Topic was to bring together various contributions based on cutting-edge computational methodologies; these include computational analysis of structures and complexes with developments and applications that integrate docking and molecular dynamics approaches, and complex experimental data such as cryogenic electron microscopy (cryo-EM).

Two articles underline the importance of new computational approaches for evaluating atomic models derived from experimental data or built *ab-initio*. The first work by Olek and Joseph dealt with the quality of models obtained by cryo-EM. In fact, the final atomic model is often incomplete or contains regions where atomic positions are less reliable or incorrectly built. They presented a software tool for the validation of the backbone trace of atomic models built in the cryo-EM maps. They use the false discovery rate analysis to segregate molecular signals from the background and show how this approach can properly complement current measures Olek and Joseph. Launay et al. tackled the challenging question of scoring in protein–protein docking. They explored several ways to perform consensus-based rescoring. They showed that rescoring performs worse than the traditional physics-based evaluation but the two complement each other and can be used in combination (Launay et al.).

Classical approaches such as molecular dynamics (MD) are useful to apprehend new biological systems. In this field, Pitard et al. studied the interaction of calmodumin (CaM) with the bacterial virulence factor, Edema Factor (EF). The system is of great interest as orthosteric and allosteric ligands have been proposed to inhibit EF activity. Using state-of-the art MD simulations, they underlined the presence of cavities at the interface between EF and CaM that could be linked to allosteric events Pitard et al.; Tang et al. combined molecular modelling and MDs to apprehend new mechanistic insights into the exciting CRISPR-Cas9 system in the DNA cleavage state. Their results provide useful guidance for engineering new CRISPR-Cas9 editing systems with improved specificity Tang et al.; Kumari et al. look at the Farnesoid X receptor (FXR) that is essential in regulating the network of genes involved in maintaining bile acid and lipid homeostasis. MDs of FXR with or without cofactors allowed a precise description of critical residue positioning during conformational changes that explain FXR activation state underlying main differences between bound and unbound forms Kumari et al.; Ghoula et al. analysed the multi domain ceramide transfer protein (CERT) implicated in the transport of ceramide from the endoplasmic reticulum to the Golgi and plays a major role in sphingolipid metabolism. Combining docking and MD simulations, the binding affinity of 14 ligands was tested. This study suggests a novel inhibitory mechanism of CERT for limonoid compounds involving the stabilization of the sub-domain interface and could help in developing new and potentially more selective inhibitors of this transporter Ghoula et al.

As previously mentioned, experimental 3D structures or structural models are crucial for the design of new drug molecules. Gheyouche et al. applied different approaches to model the structure of RHOA-ARHGEF1 complex and they further analysed the protein-protein interface. They refined the models using MD simulations and highlighted the importance of data-driven human inspection. The modelled RHOA-ARHGEF1 interface will be extremely useful for the design of inhibitors targeting this protein-protein interaction (PPI). Gheyouche et al. Similarly, Pal et al. look at systems of economic interest. They have characterized, using molecular docking, immune response molecules of duck protein TLR3, TLR7, and RIGI and predicted to have potent antiviral activities against different identified strains of Avian influenza Pal et al.; Gobinath et al. combined experiments and docking approaches in COVID research. They have screened and proposed new indole derivatives on the famous spike glycoprotein of SARS-CoV-2 Gobinath et al.

At a larger-scale, Chakraborti et al. looked at the infectious pathogen with a serious global impact: *Mycobacterium tuberculosis*. There is a constant need to search for novel therapeutic strategies because of the emergence of multidrug-resistant *tuberculosis* (MDR-TB). Universal stress protein (USP, Rv1636) is a perfect target in this field. A library of 1.9 million compounds was subjected to virtual screening, which led to the selection of 2,000 hits through an enrichment process, then 22 potential binders of Rv1636 were prioritized for experimental validations where two compounds of natural origin showed promising binding affinities Chakraborti et al.; Vedithi et al. looked at the proteome of *Mycobacterium leprae*. They presented a large set of computational approaches to unravel new potential druggable targets Vedithi et al.

Finally, Souza et al. presented innovative approaches to perform high-throughput ligand-protein docking calculations by using coarse-grained molecular dynamics simulations, based on the most recent version of the Martini force field. Their approach, characterized by excellent computational efficiency, offers a level of accuracy comparable to all-atom simulations Souza et al.; Jiang et al. looked at the Interaction of leukocyte integrin macrophage-1 antigen (Mac-1) to platelet glycoprotein Ibα (GPIbα) implicated in haemostasis and inflammatory responses. They performed a series of “ramp-clamp” steered molecular dynamics (SMD) simulations and compared the results with single molecular AFM measures. The concordance in the results underlined the importance of such approach to understand the platelet–leukocyte interaction in haemostasis and inflammatory responses under mechano- and microenvironments Jiang et al.

This special issue is dedicated to the loving memory of Prof. Narayanaswamy Srinivasan who left us too soon on the third of September 2021 (Eisenhaber et al., 2021). As a passionate scientist and a wonderful human being, he is a true inspiration. May his soul rest in peace.
